# Awakening the soul during travel: influence mechanism of memorable tourism experience on university students’ life meaning

**DOI:** 10.3389/fpsyg.2025.1521716

**Published:** 2025-03-31

**Authors:** Qiaolan Su

**Affiliations:** School of Tourism and Sport Health, Hezhou University, Hezhou, China

**Keywords:** memorable tourism, life meaning, Self-Determination Theory, positive affect, self-reflection, influence mechanisms

## Abstract

In recent years, the mental health issues of university students have received increasing attention. Research has shown that meaningful life experiences, such as memorable tourism experiences, can enhance individuals’ sense of meaning in life and promote mental health. This study explores the mechanisms through which memorable tourism experiences influence the sense of meaning in life among university students, focusing on the mediating roles of positive affect, self-reflection, and personality traits, as well as the moderating role of gender. Using a questionnaire survey, data were collected from 880 university students. Validated scales, including the “Memorable Tourism Experience Scale, ““Positive Affect Scale,” “Self-Reflection Scale,” “Personality Trait Scale,” and “Meaning in Life Scale,” were employed to measure key constructs. Data were analyzed using structural equation modeling (SEM) and multi-group analysis to test the hypothesized relationships and moderating effects. The results indicate that memorable tourism experiences have a significant positive impact on university students’ sense of meaning in life. Positive affect, self-reflection, and personality traits all partially mediate this relationship, with positive affect exhibiting the strongest mediating effect, while personality traits show relatively weaker mediation. Furthermore, gender moderates the relationship between memorable tourism experiences and positive affect, as well as the relationship between personality traits and meaning in life. These findings support Self-Determination Theory, demonstrating that memorable tourism experiences can fulfill individuals’ basic psychological needs and enhance their sense of meaning in life through multiple psychological mechanisms. This study provides practical insights for mental health interventions targeting university students, particularly highlighting the importance of reflective activities and personalized support in amplifying the psychological benefits of positive experiences.

## 1 Introduction

In recent years, with the increase in societal pressures and the accelerated pace of life, the mental health issues of university students have drawn considerable attention. According to the *Report on the Mental Health Development of Chinese Citizens (2021–2022)*, young adults are identified as a high-risk group for depression, with a detection rate of 24.1% for depressive risk among those aged 18–24, often due to perceived meaninglessness in life (Fu and Zhang, 2023). Major stress sources for university students include academic burdens, uncertainty regarding future careers, and homesickness, all closely associated with depression and anxiety (Liu et al., 2024). When students feel life is monotonous or meaningless, the risk of depression and anxiety increases (Carranza Esteban et al., 2022). As a type of positive experience that can evoke profound emotions and reflection, memorable tourism is considered an effective psychological intervention, helping individuals attain psychological growth and life meaning (Danby and Grajfoner, 2022; Kim et al., 2015; Sthapit et al., 2019). However, how tourism experiences influence students’ sense of life meaning through internal psychological mechanisms remains unclear. Particularly in the context of the diverse psychological needs of today’s university students, understanding how positive experiences can meet these needs and thereby foster psychological growth and life meaning is an issue worth deeper investigation (Nair and Otaki, 2021). Therefore, based on Self-Determination Theory, exploring the influence mechanism of memorable tourism on university students’ life meaning holds both theoretical and practical significance.

Positive affect is not merely a fleeting sense of pleasure; it also encourages individuals to adopt a more open and positive mindset when facing life’s challenges, thereby building long-term psychological resources. Câmara et al. (2022) found that memorable tourism experiences significantly enhance positive affect, which not only plays a role during the experience itself but also acts as a mediator to enhance the sense of meaning in life. Meanwhile, self-reflection, as a deep introspective process, helps individuals transform positive experiences into deeper psychological meaning. For example, Danby and Grajfoner (2022) demonstrated that self-reflection enables individuals to extract life’s purpose and meaning from tourism experiences, further strengthening their sense of meaning in life. In addition, Ryan et al. (2021) highlighted the crucial role of individual traits in the internalization of experiences. Individuals with higher personality traits, such as emotional stability and openness, are more likely to derive satisfaction and meaning from positive experiences. Engels et al. (2022) also supported this view, suggesting that individuals with high personality traits are better equipped to meet their psychological needs through memorable experiences. These findings indicate that positive affect, self-reflection, and personality traits together form a multi-layered mechanism through which memorable tourism experiences influence the sense of meaning in life.

However, existing research on the mechanisms through which memorable tourism experiences influence university students’ sense of meaning in life remains insufficient. First, although prior studies have explored the impact of tourism experiences on mental health, research on how these experiences indirectly affect the sense of meaning in life through emotional, cognitive, and individual personality traits is still not systematic (Godovykh and Tasci, 2022). Second, gender differences may play a moderating role in this process; for instance, variations in emotional responses and depth of reflection between genders may influence the effectiveness of memorable tourism experiences. However, related research remains limited (Grossman and Wood, 1993; Nolen-Hoeksema and Aldao, 2011). Finally, studies focusing on university students as a specific population are still scarce. This group is at a critical stage of psychological development, with unique psychological needs and developmental pathways distinct from other populations.

Based on the above context, this study focuses on university students and systematically explores the mechanisms through which memorable tourism experiences influence their sense of meaning in life, using Self-Determination Theory as the theoretical framework. Specifically, the study examines the mediating roles of positive affect, self-reflection, and personality traits, as well as the moderating effect of gender in this relationship. By addressing these research gaps, this study not only provides new perspectives for theoretical exploration but also offers scientific evidence for mental health education and practical interventions in higher education. The goal is to help university students achieve deeper psychological growth and a heightened sense of meaning in life within the complex social environment they navigate.

## 2 Literature review and research hypotheses

### 2.1 Basic concepts

#### 2.1.1 Memorable tourism experience

Memorable tourism experience refers to a unique, emotionally rich, and lasting memory generated during travel. This experience not only includes novel and pleasurable emotions but may also involve challenges and reflection. According to Kim et al. (2012), the essence of memorable tourism lies in its capacity to evoke profound emotional responses that lead individuals to reflect on and gain insights into life. These experiences often have a long-term impact on individuals’ lives, contributing to enhanced psychological wellbeing and life satisfaction (Knobloch et al., 2017). Participants were asked to recall and describe their most impactful tourism experiences and rate their emotional and reflective responses on a Likert scale, based on the criteria adapted from Kim et al. (2012). This approach allowed for a consistent and reliable measurement of the concept across the study.

#### 2.1.2 Life meaning

Life meaning is the individual’s understanding and experience of purpose, value, and meaning in their life. Studies indicate that individuals with a higher sense of life meaning generally exhibit stronger psychological resilience and greater life satisfaction (Steger et al., 2006). Wong (2011) argues that life meaning enables individuals to maintain a positive attitude in the face of stress and adversity, serving as an essential component of psychological health. For university students, achieving a sense of life meaning can aid in navigating various challenges, such as academic demands and career choices.

#### 2.1.3 Positive affect

Positive affect refers to the pleasurable emotional states such as joy, satisfaction, and pride experienced by individuals in specific contexts. It is one of the key indicators of subjective wellbeing in psychological research (Fredrickson, 2004). According to Fredrickson’s “Broaden-and-Build Theory, “positive affect broadens an individual’s cognitive scope, enhances their flexibility in thinking and behavior, and accumulates resources that positively influence long-term mental health. In the context of tourism, positive affect is often elicited by novel experiences, emotional interactions with others, and the enjoyment derived from natural environments (Kim et al., 2012). These emotions not only enhance an individual’s immediate sense of wellbeing but can also be internalized and transformed into a deeper understanding of life goals, thereby further enhancing their sense of meaning in life (Câmara et al., 2022). Thus, positive affect serves as a vital bridge between tourism experiences and psychological growth.

#### 2.1.4 Self-reflection

Self-reflection refers to the cognitive process in which individuals examine and analyze their experiences, behaviors, and emotional states. It is a crucial mechanism for self-improvement and inner growth (Grant et al., 2002). Through revisiting past experiences, self-reflection allows individuals to derive profound meaning and value. The diverse experiences encountered during tourism provide significant opportunities for self-reflection (Godovykh and Tasci, 2022). Studies have shown that engaging in deep reflection after tourism experiences can help individuals transform fleeting emotional responses into long-lasting psychological significance, while also enhancing their understanding of personal life goals and values (Ryan et al., 2021). For university students, self-reflection not only fosters psychological resilience but also equips them to better navigate academic and life challenges.

#### 2.1.5 Personality traits

Personality traits refer to relatively stable psychological characteristics of individuals in terms of emotions, behaviors, and cognitive patterns, commonly measured using the “Big Five Personality Traits” model. This model includes openness, extraversion, agreeableness, conscientiousness, and emotional stability (McCrae et al., 1999). Personality traits not only serve as critical psychological resources for adapting to external environments but also function as mediating mechanisms, influencing how external experiences are transformed into intrinsic psychological meaning (Jayawickreme et al., 2021). In the context of tourism, an individual’s personality traits may affect how they perceive, process, and internalize tourism experiences. For instance, individuals with high openness are more likely to engage with novel experiences during travel, deriving emotional satisfaction and psychological meaning from them (Wong, 2011). Similarly, individuals with high emotional stability tend to interpret experiences positively, remaining less affected by negative external events (Ryan et al., 2021). Moreover, personality traits can influence emotional regulation, self-awareness, and behavioral responses, enabling individuals to transform short-term tourism experiences into enduring psychological growth and a stronger sense of meaning in life (Engels et al., 2022).

### 2.2 Theoretical hypotheses

According to Self-Determination Theory, individuals’ psychological wellbeing and life meaning enhancement stem from fulfilling the three basic psychological needs: autonomy, competence, and relatedness (Ryan et al., 2021). In the context of tourism, positive affect satisfies the need for autonomy by offering individuals freedom of choice and control over their experiences; self-reflection helps individuals build competence by allowing them to recognize their growth through reflective processes; and personality traits are linked to self-awareness, with traits like openness enabling individuals to gain new insights and emotional experiences from tourism, thereby fulfilling their need for relatedness (Buckley, 2023; Ritchie and Crouch, 2003). Together, these variables contribute to the overall enhancement of life meaning.

#### 2.2.1 Memorable tourism experience and life meaning in university students

Memorable tourism experiences often involve profound emotional and cognitive engagements, fulfilling these core psychological needs. For instance, individuals typically have high freedom of choice in planning their travel, satisfying the need for autonomy (Ryan et al., 2021). Additionally, overcoming challenges during travel (such as navigating unfamiliar environments or problem-solving) enhances a sense of achievement, fulfilling the need for competence (Ritchie and Crouch, 2003). Furthermore, social interactions and cultural exchanges during travel satisfy relatedness, allowing individuals to experience emotional connections with others and the environment (Sthapit et al., 2019).

These experiential factors work together to help individuals form a positive understanding of themselves and their lives, making it easier for them to discover life’s meaning and sense of value. For instance, Kim et al. (2012) found that memorable tourism experiences provide not only short-term pleasure but also lasting psychological satisfaction, leading to a profound perception of life meaning. Based on Self-Determination Theory, this study proposes the following hypothesis:

**Hypothesis 1 (H1)**: Memorable tourism experience has a positive impact on life meaning in university students.

#### 2.2.2 The mediating role of positive affect

Psychological wellbeing and life meaning are derived from fulfilling basic psychological needs, with Positive affect playing a crucial supporting role. Memorable tourism experiences can evoke Positive affect, such as joy, satisfaction, and a sense of accomplishment, which facilitate the experience of autonomy, competence, and relatedness (Ryan et al., 2021). Positive affect not only represents an immediate emotional reaction but also supports the fulfillment of psychological needs, thereby enhancing one’s perception of life meaning.

Research has shown that Positive affect plays a key role in individuals’ internalization of experiences. Ryan et al. (2021) indicate that Positive affect enhances psychological resilience and self-efficacy, enabling individuals to derive sustained meaning from experiences. Shin et al. (2023) found that tourism experiences rich in Positive affect often strengthen individuals’ sense of life meaning by supporting the fulfillment of psychological needs. Thus, Positive affect is viewed as a crucial mediating variable in the relationship between memorable tourism and life meaning, explaining how tourism experiences enhance life meaning through Positive affect.

**Hypothesis 2a (H2a)**: Memorable tourism experience positively influences Positive affect.

**Hypothesis 2b (H2b)**: Positive affect positively influences life meaning.

**Hypothesis 2 (H2)**: Positive affect positively mediates the relationship between memorable tourism experience and life meaning.

#### 2.2.3 The mediating role of self-reflection

Following a positive experience, individuals can internalize the content and meaning of the experience through self-reflection, transforming temporary emotional experiences into lasting life meaning. Self-reflection is an introspective activity where individuals re-evaluate their feelings and behaviors from the experience, continuously redefining their relationship with the world (Ryan et al., 2021). During memorable tourism, individuals often reflect on the value and meaning of their experiences, internalizing them as a deeper understanding of life (Paatlan and Ranga, 2024).

Seggelen-Damen and Dam (2016) suggest that self-reflection enables individuals to extract a sense of value and meaning from positive experiences, enhancing their psychological wellbeing. Specifically, the reflective process after a travel experience can clarify the insights gained, thereby promoting recognition of self-worth and life meaning (Sheldon, 2020). In this study, self-reflection is seen as an important mediating variable between memorable tourism and life meaning, helping explain how tourism experiences enhance life meaning through reflection.

**Hypothesis 3a (H3a)**: Memorable tourism experience positively influences self-reflection.

**Hypothesis 3b (H3b)**: Self-reflection positively influences life meaning.

**Hypothesis 3 (H3)**: Self-reflection positively mediates the relationship between memorable tourism experience and life meaning.

#### 2.2.4 The mediating role of personality traits

Personality traits, as stable psychological characteristics of individuals, have profound impacts on their emotions, behaviors, and ways of responding to external experiences. Different dimensions of personality traits, such as openness and emotional stability, may serve as mediators between memorable tourism experiences and a sense of meaning in life. Specifically, individuals with higher levels of openness and emotional stability are more likely to experience positive emotions and engage in deep self-reflection during novel, challenging, and culturally enriching tourism experiences. These factors, in turn, enhance their perception of life’s meaning (McCrae et al., 1999).

Research indicates that individuals with high openness often exhibit a stronger desire to explore and a greater ability to embrace new experiences, allowing them to actively seize opportunities for psychological growth during tourism experiences (Kim et al., 2020; Wen and Huang, 2019). Meanwhile, individuals with high emotional stability are better equipped to maintain a positive mindset and manage their emotions when faced with uncertainties and challenges in travel, making it easier for them to derive positive emotional experiences and a sense of self-identity (Crust, 2020).

Therefore, in this study, personality traits are considered a crucial mediating variable between memorable tourism experiences and the sense of meaning in life, helping to explain how tourism experiences contribute to enhancing life meaning through personality traits. The specific hypotheses are as follows:

**Hypothesis 4a (H4a)**: Memorable tourism experiences positively influence personality traits.

**Hypothesis 4b (H4b)**: Personality traits positively influence the sense of meaning in life.

**Hypothesis 4 (H4)**: Personality traits mediate the positive relationship between memorable tourism experiences and the sense of meaning in life.

#### 2.2.5 The moderating role of gender

Gender plays a critical moderating role in psychological experiences and behavioral responses. Previous research has shown that men and women exhibit significant differences in emotional expression, reflective habits, and coping strategies, which may influence how individuals perceive and internalize memorable tourism experiences (Aggarwal, 2024). Specifically, women tend to have more sensitive and enriched emotional experiences and are more likely to achieve psychological satisfaction through emotional resonance during tourism experiences (Baqutayan et al., 2018). In contrast, men often demonstrate stronger emotional regulation and may focus more on self-reflection and overcoming challenges to enhance their sense of meaning in life during tourism (Else-Quest et al., 2012).

Gender differences may be particularly pronounced in the pathways involving positive affect and self-reflection. For instance, when experiencing memorable tourism, women often combine their experiences with emotional memories, creating intense positive emotions that can leave lasting positive emotional aftereffects, ultimately contributing to their sense of meaning in life. Men, on the other hand, may focus more on their performance during challenges and use self-reflection to enhance their understanding of their abilities and values, thereby increasing their perception of life’s meaning (Pung et al., 2024).

Additionally, gender may also influence the role of personality traits in the pathways from tourism experiences to the sense of meaning in life. Studies have shown that women often score higher on traits like openness and agreeableness, making them more likely to derive emotional resonance and a sense of belonging from novel experiences. Meanwhile, men tend to have higher emotional stability and self-efficacy, enabling them to better cope with and internalize challenging activities, thus creating different moderating effects between memorable tourism experiences and life purpose (Su et al., 2022).

Therefore, in this study, gender is considered a moderating variable that influences all pathways from memorable tourism experiences to the sense of meaning in life. The specific hypotheses are as follows:

**Hypothesis 5a (H5a)**: Gender moderates the effect of memorable tourism experiences on the sense of meaning in life.

**Hypothesis 5b (H5b)**: Gender moderates the effect of memorable tourism experiences on positive affect.

**Hypothesis 5c (H5c)**: Gender moderates the effect of memorable tourism experiences on self-reflection.

**Hypothesis 5d (H5d)**: Gender moderates the effect of memorable tourism experiences on personality traits.

**Hypothesis 5e (H5e)**: Gender moderates the effect of positive affect on the sense of meaning in life.

**Hypothesis 5f (H5f)**: Gender moderates the effect of self-reflection on the sense of meaning in life.

**Hypothesis 5g (H5g)**: Gender moderates the effect of personality traits on the sense of meaning in life.

**Figure 1:**
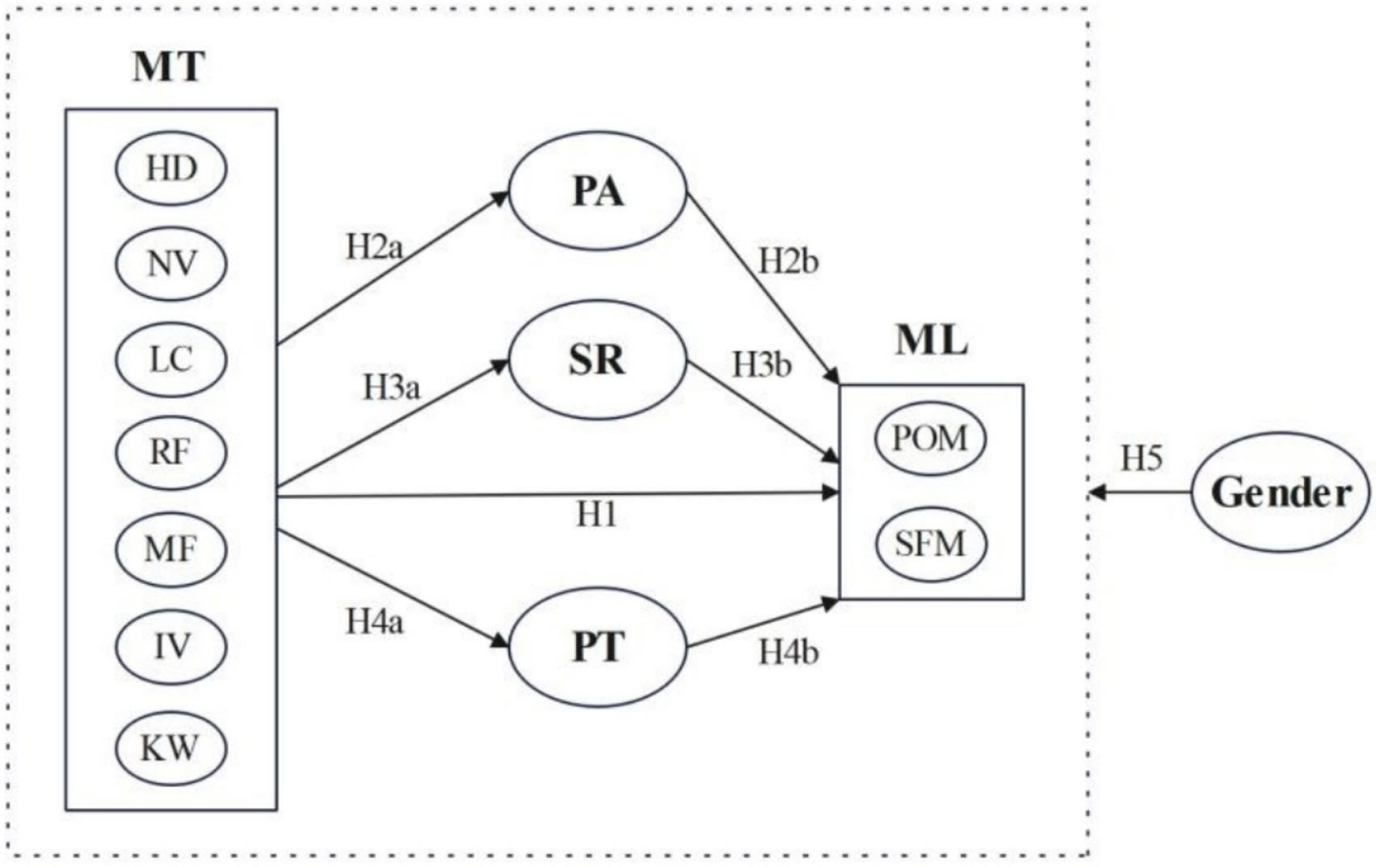
Structure model. MT, memorable tourism; PA, positive affect; SR, Self reflection; PT, personality traits; ML, meaning in life; HD, Hedonism; NV, Novelty; LC, Local culture; RF, Refreshment; MF, Meaningfulness; IV, Involvement; KW, Knowledge; POM, Presence of Meaning; SFM, Search for Meaning.

Based on the above hypotheses, this study constructed a model illustrating the relationships among university students’ memorable tourism experiences, sense of meaning in life, positive affect, self-reflection, personality traits, and gender (Figure 1).

## 3 Research design

### 3.1 Questionnaire design

The questionnaire for this study is divided into two parts: the primary content module and the demographic variables module. The primary content module encompasses five dimensions: memorable tourism experience, Positive affect, self-reflection, life meaning, and personality traits. All scales are scored using a 5-point Likert scale, where 1 indicates “strongly disagree” and 5 indicates “strongly agree.” The demographic variables include basic information about respondents, such as gender, age, major, and travel experience, to facilitate subsequent analysis of differences among various groups.

### 3.2 Variable measurement

#### 3.2.1 Memorable tourism experience scale

This study uses the Memorable Tourism Experience Scale (MTEs) developed by Kim et al. (2012) to measure respondents’ tourism experiences. This scale includes 24 items, covering dimensions such as cultural experience, novelty, emotional involvement, and social interaction. Each item is rated on a 5-point Likert scale (1 = strongly disagree, 5 = strongly agree). The MTEs scale is widely used in tourism research and has high reliability and validity.

#### 3.2.2 Positive affect scale

Positive affect is measured using the Positive Affect portion of the Positive and Negative Affect Schedule (PANAS) (Watson et al., 1988). This scale includes 10 items that assess positive emotional responses during tourism, such as “excitement” and “pleasure.” The scoring is based on a 5-point Likert scale (1 = strongly disagree, 5 = strongly agree). The PANAS scale is well-established in affective research and has been widely validated for reliability and validity.

#### 3.2.3 Self-reflection scale

Self-reflection is measured using the self-reflection portion of the Self-Reflection and Insight Scale (SRIS) (Grant et al., 2002), which includes six items measuring individuals’ capacity to reflect on their thoughts and behaviors. Each item is rated on a 5-point Likert scale (1 = strongly disagree, 5 = strongly agree). This scale has been applied in various research fields, demonstrating good reliability.

#### 3.2.4 Meaning in life scale

Life meaning is assessed using the Meaning in Life Questionnaire (MLQ) developed by Steger et al. (2006). This scale includes 10 items divided into two dimensions: the presence of life meaning and the search for life meaning. Each item is rated on a 5-point Likert scale (1 = strongly disagree, 5 = strongly agree). The MLQ is widely used in psychological research and has high reliability and validity.

#### 3.2.5 Personality traits scale

Personality traits are measured using the Big Five Inventory-10 (BFI-10) developed by Rammstedt and John (2007). This brief scale includes 10 items that assess the five dimensions of personality: extraversion, agreeableness, conscientiousness, emotional stability, and openness. Each item is rated on a 5-point Likert scale (1 = strongly disagree, 5 = strongly agree). The BFI-10 is concise and effective, suitable for large-scale surveys, with good validity and reliability.

### 3.3 Data collection and sampling

#### 3.3.1 Sampling method

This study used a convenience sampling method, targeting university students as the study population. Convenience sampling was selected due to the researchers’ collaborative relationship with Chinese universities and limitations in time and resources, allowing for the collection of sufficient and valid samples within a relatively short period (Etikan et al., 2016). This study used a convenience sampling method, which, while efficient and practical, has certain limitations, particularly in terms of the representativeness of the sample (Golzar et al., 2022). As such, the generalizability and external validity of the findings may be limited. Future studies could consider employing a more representative random sampling method to enhance the external validity of the results. Additionally, the sample in this study has specific socio-demographic characteristics, such as a majority of university students with relatively rich travel experience. These factors could influence the results. For instance, age, academic major, and travel experience may shape how individuals perceive and reflect on their tourism experiences, which could affect their sense of life meaning.

#### 3.3.2 Data collection process

The data collection process lasted from July 1, 2024, to October 1, 2024, spanning 3 months. This study conducted a survey in universities across Guangxi Zhuang Autonomous Region, China, in collaboration with university management systems and the research team. The survey included students from various majors and academic years to ensure the data reflected diverse groups’ tourism experiences and perceptions of meaning in life. The sample selection criteria included: (1) voluntary participation in the survey and (2) ensuring diversity in majors and academic years to provide a stable sample size.

The survey was administered using the platform Wenjuanxing.^1^ Researchers distributed the questionnaire links via university student information systems and social media platforms such as WeChat and QQ. The links were further disseminated through class groups and academic advisors. During the survey, the research team interacted with respondents to address any questions they encountered while completing the questionnaire. All participants were informed that their participation was entirely voluntary and that their data would remain strictly confidential and be used solely for academic research. Throughout the data collection process, the research team adhered to strict privacy protection principles, ensuring all data were anonymized to eliminate privacy concerns.

A total of 920 questionnaires were collected, resulting in 880 valid responses, with an effective response rate of 88.89%. According to Bentler (1987), the sample size should be at least 10 times the number of observed variables, and Loehlin (2004) suggested that to achieve stable results, the sample size should exceed 200. Therefore, the number of valid questionnaires collected in this study meets the requirements for analysis (Table 1).

**Table 1:** Demographic characteristics.

Title	Option	Frequency	%	(%)
Gender	Male	350	39.77	39.77
	Female	530	60.23	100
Age	Under 18	95	10.8	10.8
	19	322	36.59	47.39
	20	178	20.23	67.61
	21	159	18.07	85.68
	22	97	11.02	96.7
	Above 23	29	3.3	100
Grade	Freshman	366	41.59	41.59
	Sophomore	171	19.43	61.02
	Junior	205	23.3	84.32
	Senior	138	15.68	100
Travel times	1	259	29.43	29.43
	2	220	25	54.43
	3	211	23.98	78.41
	4	57	6.48	84.89
	5	133	15.11	100

## 4 Data analysis

### 4.1 Reliability and validity analysis

For reliability testing, this study used Cronbach’s *α* coefficient to assess the reliability of each scale, as shown in Table 2. Results indicate that the Cronbach’s *α* coefficients for all scales are above 0.8, exceeding the 0.7 threshold, demonstrating good internal consistency and reliability for the scales. In terms of validity analysis, the study assessed both convergent and discriminant validity as the main methods for evaluating validity.

**Table 2:** Reliability and validity test of the scale.

Factor	Observed variable	Coef.	Std. Error	*z*	Std. Estimate	Cronbach	C.R	AVE
Hedonism	HD1	1.000	–	–	0.879	0.922	0.926	0.760
	HD2	1.068	0.028	38.488	0.895			
	HD3	1.095	0.027	41.254	0.925			
	HD4	0.948	0.032	29.572	0.780			
Novelty	NV1	1.000	–	–	0.760	0.908	0.910	0.718
	NV2	1.078	0.040	26.862	0.847			
	NV3	1.121	0.039	29.003	0.903			
	NV4	1.112	0.040	27.850	0.872			
Local culture	LC1	1.000	–	–	0.815	0.879	0.884	0.718
	LC2	1.097	0.033	32.940	0.896			
	LC3	0.998	0.034	29.242	0.829			
Refreshment	RF1	1.000	–	–	0.833	0.913	0.915	0.728
	RF2	0.990	0.032	30.904	0.839			
	RF3	1.076	0.032	33.572	0.882			
	RF4	0.996	0.031	32.098	0.859			
Meaningfulness	MF1	1.000	–	–	0.843	0.904	0.906	0.763
	MF2	1.000	0.030	33.210	0.870			
	MF3	1.062	0.030	35.609	0.906			
Involvement	IV1	1.000	–	–	0.901	0.909	0.910	0.771
	IV2	1.001	0.025	40.640	0.893			
	IV3	0.867	0.025	35.341	0.840			
Knowledge	KW1	1.000	–	–	0.873	0.900	0.905	0.762
	KW2	1.027	0.026	39.230	0.911			
	KW3	1.079	0.033	32.832	0.833			
Positive affect	PA1	1.000	–	–	0.838	0.954	0.957	0.690
	PA10	1.107	0.036	30.740	0.824			
	PA2	1.013	0.032	31.752	0.840			
	PA3	1.131	0.034	33.254	0.863			
	PA4	0.957	0.047	20.413	0.618			
	PA5	1.004	0.031	32.306	0.849			
	PA6	1.126	0.033	33.662	0.869			
	PA7	1.188	0.034	35.105	0.890			
	PA8	1.139	0.036	31.223	0.831			
	PA9	1.111	0.034	32.685	0.854			
Self-reflection	SR1	1.000	–	–	0.800	0.945	0.945	0.742
	SR2	1.086	0.037	29.540	0.850			
	SR3	1.102	0.035	31.084	0.880			
	SR4	1.092	0.034	31.822	0.894			
	SR5	1.077	0.034	31.975	0.897			
	SR6	1.016	0.035	29.200	0.843			
Presence of meaning	POM1	1.000	–	–	0.893	0.914	0.918	0.695
	POM2	1.016	0.026	39.173	0.890			
	POM3	0.996	0.029	34.188	0.835			
	POM4	1.031	0.026	39.309	0.891			
	POM5	0.663	0.031	21.433	0.628			
Search for meaning	SFM1	1.000	–	–	0.794	0.938	0.939	0.755
	SFM2	1.060	0.036	29.067	0.850			
	SFM3	1.127	0.036	31.413	0.898			
	SFM4	1.129	0.035	31.932	0.909			
	SFM5	1.117	0.036	30.963	0.889			
Personal trait	PT1	1.000	–	–	0.467	0.847	0.846	0.361
	PT10	1.865	0.143	13.037	0.743			
	PT2	1.238	0.110	11.291	0.548			
	PT3	1.029	0.095	10.782	0.506			
	PT4	1.727	0.137	12.612	0.685			
	PT5	0.976	0.089	10.916	0.517			
	PT6	1.979	0.153	12.967	0.732			
	PT7	1.121	0.096	11.730	0.589			
	PT8	1.581	0.126	12.507	0.672			
	PT9	0.810	0.080	10.144	0.458			

The evaluation of convergent validity followed the recommendations of Fornell and Larcker (1981), examining standardized factor loadings, composite reliability (CR), and average variance extracted (AVE) as indicators. As shown in the table, all scales have standardized loadings (*λ*) greater than 0.7, with t-values significant at the *p* < 0.001 level, meeting the standard of loadings above 0.6, indicating high measurement reliability. Additionally, the CR values for all constructs exceed 0.8, aligning with the acceptable threshold suggested by Hayes (2017) (>0.7), indicating good internal consistency within each construct. Moreover, the AVE values are all above 0.5, meeting Fornell and Larcker (1981) ideal standard, suggesting that the constructs can adequately explain the variance of the measured variables, thus supporting good convergent validity.

For discriminant validity, following Fornell and Larcker (1981) criterion, the square root of the AVE of each latent variable should be greater than its correlation with other latent variables. The results, as shown in Table 3, indicate that the square root of the AVE for each latent variable exceeds its correlations with other latent variables, demonstrating satisfactory discriminant validity. Therefore, the latent variables in this study exhibit strong discriminant validity, supporting the structural validity of the model.

**Table 3:** Discriminant validity test of latent variables.

	Hedonism	Novelty	Local culture	Refreshment	Meaningfulness	Involvement	Knowledge	Positive affect	Self-reflection	Presence of meaning	Search for meaning	Personal trait
Hedonism	**0.872**											
Novelty	0.781	**0.847**										
Local culture	0.785	0.824	**0.847**									
Refreshment	0.775	0.78	0.849	**0.853**								
Meaningfulness	0.735	0.732	0.79	0.825	**0.874**							
Involvement	0.724	0.787	0.81	0.798	0.82	**0.878**						
Knowledge	0.731	0.744	0.788	0.819	0.798	0.834	**0.873**					
Positive affect	0.761	0.753	0.807	0.837	0.819	0.82	0.852	**0.831**				
Self-reflection	0.527	0.585	0.602	0.638	0.613	0.645	0.643	0.707	**0.861**			
Presence of meaning	0.618	0.598	0.674	0.675	0.682	0.703	0.679	0.76	0.65	**0.834**		
Search for meaning	0.482	0.482	0.524	0.519	0.523	0.571	0.526	0.595	0.527	0.659	**0.869**	
Personal trait	0.331	0.322	0.406	0.395	0.364	0.399	0.384	0.445	0.371	0.388	0.405	**0.601**

The bold diagonal values represent the square root of AVE, and the lower triangle shows the Pearson correlations between dimensions.

### 4.2 Model fit testing

Model fit reflects the extent to which the theoretical model aligns with the actual data (Byrne, 2010). To optimize model fit, this study used the modification index method, making adjustments to residuals with high correlation to improve fit indices such as the Goodness of Fit Index (GFI) (Baumgartner and Homburg, 1996). The single-factor test by Harman (1976) shows that the total variance explained by all factors in this study is 77.96%, with the variance explained by the first factor at 49.96%, below the 50% threshold. This indicates that no single factor explains the majority of variance, suggesting that common method bias is not significant in this study. Additionally, following Podsakoff et al. (2003), a common method factor was incorporated in AMOS during confirmatory factor analysis to control for potential common method bias, and the fit indices for the adjusted model were evaluated.

A comparison of fit indices across different factor models shows that the five-factor model displays good fit, supporting its validity. As shown in Table 4, the *χ*^2^/df value is 3.548, which falls within the ideal range of 1–3, indicating a reasonable model-data fit. The SRMR is 0.038, and the RMSEA is 0.054, both below the commonly accepted threshold of 0.08, suggesting that model error is within an acceptable range. Other fit indices include a GFI of 0.85, which, while slightly below the ideal value of 0.90, is still considered acceptable in certain contexts (Baumgartner and Homburg, 1996). Additional indices, such as AGFI (0.825), IFI (0.942), CFI (0.942), and TLI (0.935), meet or exceed the 0.90 threshold, indicating that the overall model fit is satisfactory.

**Table 4:** Model fit indices.

Common indicators	*χ* ^2^	df	*p*	*χ*^2^/df	GFI	RMSEA	RMR	CFI	NFI	NNFI
Evaluation criteria	–	–	>0.05	<3	>0.9	<0.10	<0.05	>0.9	>0.9	>0.9
Value	3,274.353	923	0	3.548	0.85	0.054	0.027	0.942	0.921	0.935
Other indicators	TLI	AGFI	IFI	PGFI	PNFI	PCFI	SRMR	RMSEA 90% CI		
Evaluation criteria	>0.9	>0.9	>0.9	>0.5	>0.5	>0.5	<0.1	–		
Value	0.935	0.825	0.942	0.726	0.821	0.84	0.038	0.048–0.056		

### 4.3 Path effect test

This study utilized the Bootstrap procedure (original sample = 880, bootstrap samples = 5,000) to estimate the path coefficients and significance levels of the model, as shown in Table 5.

**Table 5:** Path effect test.

Assumption	Path	Non-standardized coefficient	SE	*Z* value	*p*	Standardized coefficient	Hypothesis testing
H1	MT → ML	0.638	0.021	30.421	0	0.716	Set up
H2a	MT → PA	0.919	0.016	58.010	0	0.891	Set up
H2b	PA → ML	0.322	0.043	7.457	0	0.373	Set up
H3a	MT → SR	0.735	0.028	26.695	0	0.669	Set up
H3b	SR → ML	0.172	0.025	7.001	0	0.213	Set up
H4a	MT → PT	0.372	0.028	13.258	0	0.408	Set up
H4b	PT → ML	0.107	0.023	4.613	0	0.110	Set up

The results reveal that memorable tourism experiences have a robust, direct positive impact on life meaning (standardized coefficient = 0.716, *Z* = 30.421, *p* < 0.01), underscoring the central role of such experiences in enhancing students’ perceptions of life meaning.

Furthermore, memorable tourism experiences significantly influence several key mediators. Specifically, they exert a strong positive effect on positive affect (*β* = 0.891, *Z* = 58.010, *p* < 0.01) and on self-reflection (*β* = 0.669, *Z* = 26.695, *p* < 0.01), as well as a moderate impact on personality traits (*β* = 0.408, *Z* = 13.258, *p* < 0.01). In turn, these mediators contribute to life meaning: positive affect directly influences life meaning (*β* = 0.373, *Z* = 7.457, *p* < 0.01) and self-reflection also exerts a significant positive effect (*β* = 0.460, *Z* = 14.915, *p* < 0.01). Although the effect of personality traits on life meaning is relatively smaller (*β* = 0.110, *Z* = 4.613, *p* < 0.01), it remains statistically significant.

Collectively, these findings suggest that memorable tourism experiences not only enhance life meaning directly but also do so indirectly by fostering positive emotional responses and reflective processes. This multifaceted pathway highlights the critical role of both affective and cognitive mechanisms in the formation of a meaningful life, supporting the broader theoretical framework of the study.

### 4.4 Mediation effect test

This study employed the SPSS PROCESS macro developed by Hayes (2017) to examine the mediation effects of Positive Affect (PA), Self-Reflection (SR), and Personality Traits (PT) on the relationship between Memorable Tourism Experience (MT) and Meaning in Life (ML). The results, presented in Table 6, reveal the following findings:

**Table 6:** Results of the mediation effect.

Path	Total effect	a	b	a*b	a*b (Boot SE)	a*b (*z*)	a*b (*p*)	a*b (95% BootCI)	Direct effect	Percentage	Result
MT → PA → ML	0.638**	0.919**	0.322**	0.296	0.053	5.605	0.000	0.225–0.435	0.175**	46.389%	Partial mediation
MT → SR → ML	0.638**	0.735**	0.172**	0.127	0.029	4.309	0.000	0.087–0.204	0.175**	19.864%	Partial mediation
MT → PT → ML	0.638**	0.372**	0.107**	0.04	0.014	2.938	0.003	0.022–0.075	0.175**	6.259%	Partial mediation

First, MT has a significant direct positive effect on ML (*β* = 0.175, *p* < 0.001), confirming its central role in enhancing life meaning. Importantly, the mediation analysis demonstrates that the pathway through Positive Affect is particularly robust, accounting for 46.389% of the total effect. This indicates that the immediate positive emotional responses elicited by tourism experiences are critical in shaping an individual’s perception of life meaning.

Furthermore, Self-Reflection mediates 19.864% of the total effect. This suggests that beyond the initial emotional impact, the process of introspection and cognitive evaluation plays a vital role in consolidating and deepening the meaning derived from tourism experiences. Although Personality Traits mediate a smaller portion of the effect (6.259%), their significant contribution underscores the importance of individual differences in how these experiences are internalized and translated into life meaning.

To validate the robustness of these mediation effects, a Bootstrap analysis with 1,000 resamples was performed. The indirect effect through PA was 0.296 (95% CI [0.225, 0.345]), through SR was 0.127 (95% CI [0.087, 0.204]), and through PT was 0.040 (95% CI [0.022, 0.075]).

Collectively, these findings suggest that memorable tourism experiences enhance life meaning through a multifaceted process: by triggering immediate positive emotions, fostering deeper self-reflection, and, to a lesser extent, interacting with individual personality characteristics. This integrated mediation framework not only confirms our hypotheses but also highlights the complex interplay of affective and cognitive processes in the formation of a meaningful life.

### 4.5 Gender as a moderator

To examine the moderating effect of gender on model pathways, this study employed a multi-group model comparison method. The sample was divided into male (*n* = 330) and female (*n* = 530) groups. First, an unconstrained baseline model was established without any parameter restrictions. Next, equality constraints were incrementally applied to the regression coefficients of different pathways to form constrained models. Finally, chi-square (*χ*^2^) values of the constrained models were compared with those of the baseline model. If the *χ*^2^ value of the constrained model was significantly higher than that of the baseline model, it indicated a significant moderating effect of gender on the specific pathway; otherwise, no significant moderating effect was observed (Byrne, 1993).

As shown in Table 7, compared with the baseline model, the partially constrained model revealed significant differences in the pathways “MT → PA” and “PT → ML” (∆*χ*^2^ = 5.856 and 5.453, respectively, *p* < 0.05). However, other pathways, such as “MT → ML,” “MT → SR,” “PA → ML,” and “SR → ML,” did not show significant differences in *χ*^2^ values (*p* > 0.05). These findings indicate that gender significantly moderates the pathways “Memorable Tourism Experience → Positive Affect” and “Personality Traits → Meaning in Life,” supporting hypotheses H5b and H5g. Hypotheses H5a, H5c, H5d, H5e, and H5f were not supported, indicating that gender does not significantly moderate these pathways.

**Table 7:** Test of gender moderation effects.

Model	∆*χ*^2^	*p*	∆df
Basic model			
Constrained model: MT → ML	0.186	0.666	1
Constrained model: MT → PA	5.856	0.016**	1
Constrained model: MT → SR	0.777	0.378	1
Constrained model: MT → PT	0.014	0.904	1
Constrained model: PA → ML	0.802	0.370	1
Constrained model: SR → ML	0.714	0.398	1
Constrained model: PT → ML	5.453	0.020**	1

*p < 0.05; **p < 0.01; ***p < 0.001.

Further comparisons of pathway coefficients between gender groups (see Table 8) revealed the following:

**Table 8:** Comparison for moderating effects of gender.

Path	Male		Female	
	Std. coefficients	*t*-value	Std. coefficients	*t*-value
MT → ML	0.055	0.472	0.121	1.121
MT → PA	0.917***	19.382	0.931***	16.956
MT → SR	0.725***	13.816	0.695***	14.141
MT → PT	0.494***	7.423	0.302***	6.323
PA → ML	0.491***	4.586	0.443***	4.255
SR → ML	0.176**	3.072	0.276***	5.958
PT → ML	0.160***	3.318	0.063*	1.887

*p < 0.05; **p < 0.01; ***p < 0.001.

For the pathway “MT → PA,” the coefficient for the male group was 0.917 (*t* = 19.382), while for the female group, it was 0.931 (*t* = 16.956), indicating that females are slightly more likely to derive positive affect from memorable tourism experiences, although the difference is small.

For the pathway “PT → ML,” the coefficient for the male group was 0.160 (*t* = 3.318), while for the female group, it was 0.063 (*t* = 1.887), indicating that the impact of personality traits on meaning in life is more significant among males.

In summary, gender significantly moderates the pathways “Memorable Tourism Experience → Positive Affect” and “Personality Traits → Meaning in Life.” Specifically, females are more likely to derive positive affect from memorable tourism experiences, while males are more inclined to enhance their meaning in life through personality traits. These findings suggest that psychological interventions and experiential optimizations can be tailored to the characteristics of different genders. For instance, enriching emotional experiences may better enhance females’ wellbeing, whereas focusing on the development of personality traits may be more effective for males in strengthening their sense of meaning in life.

Men and women exhibit different responses in tourism experiences, which are closely related to social cultural backgrounds, gender role expectations, and psychological responses. According to social psychology theories, the socialization process of gender roles affects emotional expression and response styles. Women tend to show more dependence and emotional communication in social interactions (Helgeson, 2017). Therefore, women may be more likely to achieve life meaning through social interactions and connections with nature, while men might focus more on self-challenge and enhancing competence. This difference indicates that gender plays a complex moderating role in memorable tourism experiences, which requires further exploration of the underlying socio-cultural and psychological mechanisms.

## 5 Conclusions and discussion

### 5.1 Research conclusions

This study focused on university students to explore the multi-level influence mechanisms of memorable tourism experiences on meaning in life, with a particular emphasis on the mediating roles of positive affect, self-reflection, and personality traits, as well as the moderating role of gender. Using structural equation modeling, the following key conclusions were drawn:

Firstly, memorable tourism experience has a significant positive impact on life meaning among university students. As a positive psychological experience, memorable tourism enables students to gain emotional fulfillment and cognitive inspiration through exploring the external world. The study shows that emotionally and cognitively enriching experiences during tourism enhance students’ understanding of life, providing them with a deeper sense of intrinsic value and purpose. This finding aligns with the studies by Kim et al. (2015) and Sthapit and Coudounaris (2018), further validating that tourism, as a positive psychological resource, can stimulate the pursuit of life meaning, enhancing psychological wellbeing. This discovery highlights the unique role of memorable tourism experiences in students’ psychological growth and provides new empirical support for the positive effects of meaningful experiences on mental health.

Positive affect acts as a vital emotional response that enables individuals to broaden their cognitive scope and develop lasting psychological resources. In the context of this study, positive affect significantly mediates the relationship between memorable tourism experiences and meaning in life, aligning with the broaden-and-build theory of positive emotions (Fredrickson, 2004). This theory highlights how positive emotions, such as joy and excitement, can expand an individual’s cognitive and behavioral repertoire, contributing to psychological growth. Empirical studies corroborate the finding that positive affect is a crucial mechanism in enhancing wellbeing and meaning in life. For instance, Câmara et al. (2022) emphasized that engaging, emotionally rich tourism experiences elicit strong positive emotions, which subsequently foster a deeper understanding of life’s purpose. Similarly, Shin et al. (2023) demonstrated that positive emotional responses in smart tourism contexts significantly predict tourists’ satisfaction and their perceptions of life meaning. In this study, the mediating role of positive affect highlights its capacity to transform short-term emotions elicited during tourism into sustained cognitive benefits, thus affirming its centrality in the interplay between memorable tourism experiences and meaning in life. However, as noted by Ryan et al. (2021), the transient nature of emotions necessitates further cognitive processing to solidify their long-term impact.

Self-reflection serves as a deep introspective process where individuals analyze and derive meaning from their past experiences. This study confirms that self-reflection partially mediates the relationship between memorable tourism experiences and meaning in life, underscoring its role in transforming transient emotional responses into enduring psychological growth. The significance of self-reflection is consistent with prior research. Danby and Grajfoner (2022) revealed that tourism experiences coupled with reflective activities can lead to profound psychological insights and an enhanced sense of life purpose. Similarly, Paatlan and Ranga (2024) found that reflective practices following impactful experiences enable individuals to extract deeper values and align their experiences with long-term life goals. This study expands on existing literature by emphasizing that self-reflection not only enhances understanding of personal experiences but also serves as a bridge between experiential events and higher-order psychological outcomes. Encouraging reflection during or after tourism activities can thus help individuals integrate these experiences into their life narratives, amplifying their long-term impact on meaning in life.

Personality traits played a significant role as partial mediators between memorable tourism experiences and meaning in life, further revealing how personality traits influence individuals’ internalization of tourism experiences. The findings demonstrated that memorable tourism experiences positively predict personality traits, such as openness and emotional stability, where individuals with higher levels of these traits are more likely to develop positive psychological states and a profound understanding of life meaning when encountering novelty and challenges during tourism. These results align with the study by Jayawickreme et al. (2021), supporting the importance of personality traits as psychological resources in personal growth. Additionally, Wong (2011) highlighted that openness and emotional stability not only enhance individuals’ emotional regulation capabilities but also enable them to more effectively derive meaning from experiences in complex environments, further supporting the findings of this study. Similarly, Godovykh and Tasci (2022) emphasized the critical role of personality traits in internalizing the effects of tourism experiences. Particularly in contexts requiring cognitive and emotional challenges, individuals with stable personality traits tend to benefit more from these experiences. This study provides a novel perspective on how individual differences influence the psychological outcomes of tourism experiences, while also underscoring the importance of personality traits in psychological intervention practices.

Gender demonstrated a significant moderating effect on the relationship between memorable tourism experiences and meaning in life. Through multi-group analysis, this study revealed that gender significantly moderated specific pathways, particularly the paths of “memorable tourism experiences → positive affect” and “personality traits → meaning in life.” Specifically, compared to males, females were more likely to derive enriched positive affect from memorable tourism experiences. This may be attributed to females’ higher sensitivity to emotional expression and resonance, enabling them to internalize novelty and social interactions during tourism as positive emotional experiences. This finding aligns with the study by Baqutayan et al. (2018), which suggests that females’ advantages in emotional experiences significantly enhance the psychological satisfaction and meaning in life gained from tourism. Similarly, Câmara et al. (2022) supported this perspective, arguing that females tend to actively express and internalize emotions during experiences, providing a theoretical basis for their ability to derive meaning from positive experiences. Regarding the path of “personality traits → meaning in life,” the study found that the path coefficient for males was significantly higher than for females. This indicates that males are more inclined to translate personality traits (e.g., emotional stability) into a profound understanding of life meaning. Males’ goal-oriented behavior and tendency to focus on overcoming challenges during tourism may enhance their capacity to internalize experiences and elevate their meaning in life. Additionally, Ryan et al. (2021) suggested that gender differences might stem from varying preferences for fulfilling self-determination needs. Females are more likely to achieve psychological growth through relational connectedness and emotional bonds, whereas males rely more on satisfying autonomy and competence needs. This theoretical perspective aligns with the findings of this study. These results further illustrate the critical role of gender differences in the internalization and psychological transformation of tourism experiences, highlighting the importance of considering gender-specific pathways in designing tourism-related interventions and experiences.

Beyond these established relationships, our findings prompt a broader reflection on the construct of life meaning. Although our study has traditionally conceptualized life meaning as the intrinsic value and purpose derived from memorable tourism experiences, recent models of psychological wellbeing suggest a more nuanced framework. For example, the concept of psychological richness—which emphasizes the complexity, novelty, and personal growth emerging from diverse life experiences (Oishi and Westgate, 2022)—extends the conventional view of wellbeing. In our research, memorable tourism experiences not only provided immediate emotional benefits and cognitive inspiration but also fostered deeper self-reflection, potentially contributing to a psychologically rich life. Emerging studies indicate that psychological richness interacts with positive psychological dimensions such as resilience and mindfulness (Mauro et al., 2025; Oishi et al., 2021). While gender-specific socialization processes might shape how individuals integrate complex experiences into their self-narratives, thereby influencing how they experience psychological richness, existing research suggests that psychological richness itself is not inherently gender-dependent (Oishi et al., 2020).

### 5.2 Practical implications

Universities and social organizations can create memorable tourism experiences for students by planning engaging and meaningful travel activities. For example, activities such as cross-cultural exchange programs, themed nature explorations, or extreme challenges can not only evoke emotional resonance but also provide students with profound insights and a sense of value during their journeys (Reis et al., 2023). Additionally, the content of these trips should emphasize diversity and personalization to cater to students’ varied interests. For instance, designing theme-based travel experiences aligned with students’ academic backgrounds or personal interests can help them not only enjoy novelty and fun but also transform their travel experiences into inspiration for future life and career planning.

Universities can organize activities that foster emotional resonance, such as cultural interactions with local residents, experiential learning projects, or team collaboration challenges, to enhance positive affect during the travel process (Rodríguez-Jiménez et al., 2022). Furthermore, post-travel activities such as sharing sessions or video recaps can extend the emotional resonance effect, reinforcing the role of positive affect in promoting psychological wellbeing. Encouraging students to engage in deep self-reflection by writing travel journals, participating in group reflection discussions, or creating personal travel reports can further help them extract deeper meaning from their travel experiences and enhance their understanding of life goals and values (Paatlan and Ranga, 2024). For students with lower levels of openness or emotional stability, emotional management and psychological resilience training can strengthen their mental resources (Reis et al., 2023). For instance, activities designed for students with low emotional stability could include stress-relief sessions, such as meditation practices or psychological support groups, to help them better manage negative emotions and fully utilize the positive effects of their travel experiences.

Universities should also consider gender differences when designing psychological interventions and experiential learning projects (Dekker et al., 2020). For example, emotional expression training and social experiences can foster psychological growth among female students, while challenging tasks and goal-oriented activities can stimulate the potential of male students. Such gender-sensitive designs can better address the distinct psychological needs of male and female students, ultimately enhancing their meaning in life. These tailored approaches not only optimize the effectiveness of tourism experiences but also provide universities with more targeted practical strategies for supporting student mental health initiatives.

### 5.3 Research limitations and future directions

Although this study has made valuable contributions to understanding the influence mechanism of memorable tourism experiences on life meaning among university students, it also has certain limitations that future research could address and expand upon:

**Sample limitations**: This study’s sample is limited to university students, which may restrict the generalizability of the findings. The psychological developmental characteristics, living environments, and social roles of university students are relatively unique, so the impact of memorable tourism experiences on life meaning might not fully apply to other age or occupational groups. Future studies could broaden the sample to include individuals from different age groups and professional backgrounds to verify the applicability of these findings across more diverse populations.

**Cross-sectional design limitation**: This study employs a cross-sectional design, which does not reveal causal relationships between variables. Although the results demonstrate significant associations among memorable tourism experience, self-reflection, Positive affect, personality traits, and life meaning, the causality cannot be confirmed. For instance, does a higher sense of life meaning influence individuals’ interpretation of tourism experiences? Future research could consider longitudinal studies to track changes within the same group, providing a clearer understanding of causality between variables.

**Self-report data limitation**: Data collection in this study primarily relied on self-report questionnaires, which may introduce subjective biases. Variables such as self-reflection and Positive affect are especially susceptible to social desirability and recall bias, potentially affecting the objectivity of the data. Future research could integrate other data collection methods, such as behavioral observations, diary studies, or physiological indicators, to obtain more comprehensive and objective data support.

## Data Availability

The original contributions presented in the study are included in the article/supplementary material, further inquiries can be directed to the corresponding author.

## Footnotes

^1^ www.wjx.cn

## References

[ref1] Aggarwal A. (2024). “Examining emotional resilience”: a comparative analysis of gender differences in emotional strength between women and men. Libr. Prog. Int. 44, 22653–22666. doi: 10.13140/RG.2.2.25392.57607

[ref2] Baqutayan S. M. S. Gul M. Ghafar S. (2018). A study of gender differences on stress and emotional intelligence. J. Adv. Res. Soc. Behav. Sci. 13, 54–65.

[ref3] Baumgartner H. Homburg C. (1996). Applications of structural equation modeling in marketing and consumer research: a review. Int. J. Res. Mark. 13, 139–161. doi: 10.1016/0167-8116(95)00038-0

[ref4] Bentler P. M. Chou C. P. (1987). Practical issues in structural modeling. Sociol. Methods Res. 16, 78–117.

[ref5] Buckley R. (2023). Tourism and mental health: foundations, frameworks, and futures. J. Travel Res. 62, 3–20. doi: 10.1177/00472875221087669

[ref6] Byrne B. M. (1993). The Maslach burnout inventory: testing for factorial validity and invariance across elementary, intermediate and secondary teachers. J. Occup. Organ. Psychol. 66, 197–212. doi: 10.1111/j.2044-8325.1993.tb00532.x

[ref7] Byrne B. M. (2010). Structural equation modeling with AMOS: basic concepts, applications, and programming (multivariate applications series), vol. 396. New York: Taylor & Francis Group, 7384.

[ref8] Câmara E. Pocinho M. Agapito D. Jesus S. N. D. (2022). Positive psychology, well-being, and mindfulness: a successful partnership towards the development of meaningful tourist experiences. J. Tour. Sustain. Well-being 10, 21–38. doi: 10.34623/m7xe-a661

[ref9] Carranza Esteban R. F. Mamani-Benito O. Caycho-Rodriguez T. Lingán-Huamán S. K. Ruiz Mamani P. G. (2022). Psychological distress, anxiety, and academic self-efficacy as predictors of study satisfaction among Peruvian university students during the COVID-19 pandemic. Front. Psychol. 13:809230. doi: 10.3389/fpsyg.2022.809230, PMID: 35548489 PMC9085258

[ref11] Crust L. (2020). Personality and mountaineering: a critical review and directions for future research. Personal. Individ. Differ. 163:110073. doi: 10.1016/j.paid.2020.110073

[ref12] Danby P. Grajfoner D. (2022). Human–equine tourism and nature-based solutions: exploring psychological well-being through transformational experiences. J. Hospital. Tour. Res. 46, 607–629. doi: 10.1177/1096348020978555

[ref13] Dekker I. De Jong E. M. Schippers M. C. De Bruijn-Smolders M. Alexiou A. Giesbers B. (2020). Optimizing students’ mental health and academic performance: AI-enhanced life crafting. Front. Psychol. 11:1063. doi: 10.3389/fpsyg.2020.01063, PMID: 32581935 PMC7286028

[ref14] Else-Quest N. M. Higgins A. Allison C. Morton L. C. (2012). Gender differences in self-conscious emotional experience: a meta-analysis. Psychol. Bull. 138, 947–981. doi: 10.1037/a0027930, PMID: 22468881

[ref15] Engels E. S. Reimers A. K. Pickel M. Freund P. A. (2022). Personality traits moderate the relationships between psychological needs and enjoyment of physical activity. Psychol. Sport Exerc. 61:102197. doi: 10.1016/j.psychsport.2022.102197

[ref16] Etikan I. Musa S. A. Alkassim R. S. (2016). Comparison of convenience sampling and purposive sampling. Am. J. Theor. Appl. Stat. 5, 1–4. doi: 10.11648/j.ajtas.20160501.11

[ref17] Fornell C. Larcker D. F. (1981). Evaluating structural equation models with unobservable variables and measurement error. J. Mark. Res. 18, 39–50. doi: 10.1177/002224378101800104

[ref18] Fredrickson B. L. (2004). The broaden–and–build theory of positive emotions. Philos. Trans. Roy. Soc. London Ser. B Biol. Sci. 359, 1367–1378. doi: 10.1098/rstb.2004.1512, PMID: 15347528 PMC1693418

[ref19] Fu X. Zhang K. (2023). China national mental health development report (2021~2022). Peijin: Social Science Literature Publishing House.

[ref20] Godovykh M. Tasci A. D. (2022). Emotions, feelings, and moods in tourism and hospitality research: conceptual and methodological differences. Tour. Hosp. Res. 22, 247–253. doi: 10.1177/1467358421103986

[ref21] Golzar J. Noor S. Tajik O. (2022). Convenience sampling. Int. J. Educ. Lang. Stud. 1, 72–77. doi: 10.22034/ijels.2022.162981

[ref22] Grant A. M. Franklin J. Langford P. (2002). The self-reflection and insight scale: a new measure of private self-consciousness. Soc. Behav. Personal. Int. J. 30, 821–835. doi: 10.2224/sbp.2002.30.8.821

[ref23] Grossman M. Wood W. (1993). Sex differences in intensity of emotional experience: a social role interpretation. J. Pers. Soc. Psychol. 65, 1010–1022. doi: 10.1037/0022-3514.65.5.1010, PMID: 8246109

[ref24] Harman H. H. (1976). Modern factor analysis (3rd edn.). Chicago, IL: University of Chicago Press.

[ref25] Hayes A. F. (2017). Introduction to mediation, moderation, and conditional process analysis: a regression-based approach (2nd edn.). New York, NY: Guilford Publications.

[ref9003] Helgeson V. S. (2017). Psychology of gender (5th edn.). New York, NY: Routledge.

[ref26] Jayawickreme E. Infurna F. J. Alajak K. Blackie L. E. Chopik W. J. Chung J. M.. (2021). Post-traumatic growth as positive personality change: challenges, opportunities, and recommendations. J. Pers. 89, 145–165. doi: 10.1111/jopy.12591, PMID: 32897574 PMC8062071

[ref27] Kim M. J. Lee C.-K. Jung T. (2020). Exploring consumer behavior in virtual reality tourism using an extended stimulus-organism-response model. J. Travel Res. 59, 69–89. doi: 10.1177/00472875188189

[ref28] Kim H. Lee S. Uysal M. Kim J. Ahn K. (2015). Nature-based tourism: motivation and subjective well-being. J. Travel Tour. Mark. 32, S76–S96. doi: 10.1080/10548408.2014.997958

[ref29] Kim J.-H. Ritchie J. B. McCormick B. (2012). Development of a scale to measure memorable tourism experiences. J. Travel Res. 51, 12–25. doi: 10.1177/0047287510385467

[ref30] Knobloch U. Robertson K. Aitken R. (2017). Experience, emotion, and eudaimonia: a consideration of tourist experiences and well-being. J. Travel Res. 56, 651–662. doi: 10.1177/0047287516650937

[ref31] Liu C. Xie Y. Xu Y. Song Z. Tang J. Shen J.. (2024). Assessing the stress-relief impact of an art-based intervention inspired by the broaden-and-build theory in college students. Front. Psychol. 15:1324415. doi: 10.3389/fpsyg.2024.1324415, PMID: 38356766 PMC10864434

[ref32] Loehlin J. C. (2004). Latent variable models: an introduction to factor, path, and structural equation analysis (4th edn.). New York, NY: Psychology Press.

[ref33] Mauro F. Di Trani M. Simione L. (2025). The psychologically rich life questionnaire: Italian validation and exploration of its relationships with mindfulness, self-compassion, and cognitive fusion within the health psychology framework. Front. Psychol. 16:1525300. doi: 10.3389/fpsyg.2025.1525300, PMID: 40051408 PMC11883556

[ref10] McCrae R. R. Costa Jr P. T. Pervin L. A. John O. P. (1999). A five-factor theory of personality handbook of personality: Theory and research, New York, NY, US: Guilford Press. pp. 139–153.

[ref34] Nair B. Otaki F. (2021). Promoting university students' mental health: a systematic literature review introducing the 4m-model of individual-level interventions. Front. Public Health 9:699030. doi: 10.3389/fpubh.2021.699030, PMID: 34249852 PMC8267876

[ref35] Nolen-Hoeksema S. Aldao A. (2011). Gender and age differences in emotion regulation strategies and their relationship to depressive symptoms. Personal. Individ. Differ. 51, 704–708. doi: 10.1016/j.paid.2011.06.012

[ref36] Oishi S. Choi H. Koo M. Galinha I. Ishii K. Komiya A.. (2020). Happiness, meaning, and psychological richness. Affect. Sci. 1, 107–115. doi: 10.1007/s42761-020-00011-z, PMID: 36042966 PMC9383031

[ref37] Oishi S. Choi H. Liu A. Kurtz J. (2021). Experiences associated with psychological richness. Eur. J. Personal. 35, 754–770. doi: 10.1177/0890207020962334

[ref38] Oishi S. Westgate E. C. (2022). A psychologically rich life: beyond happiness and meaning. Psychol. Rev. 129, 790–811. doi: 10.1037/rev0000317, PMID: 34383524

[ref39] Paatlan S. Ranga J. (2024). “Exploring sustainable tourism practices for fostering meaningful travel experiences: a global perspective” in Sustainable tourism, part a: balancing conservation and progress in a dynamic industry (Bingley, UK: Emerald Publishing Limited), 183–196.

[ref40] Podsakoff P. M. MacKenzie S. B. Lee J.-Y. Podsakoff N. P. (2003). Common method biases in behavioral research: a critical review of the literature and recommended remedies. J. Appl. Psychol. 88, 879–903. doi: 10.1037/0021-9010.88.5.879, PMID: 14516251

[ref41] Pung J. M. Khoo C. Del Chiappa G. Lee C. (2024). Tourist transformation: an empirical analysis of female and male experiences. Tour. Recreat. Res. 49, 1036–1050. doi: 10.1080/02508281.2022.2117353

[ref42] Rammstedt B. John O. P. (2007). Measuring personality in one minute or less: a 10-item short version of the big five inventory in English and German. J. Res. Pers. 41, 203–212. doi: 10.1016/j.jrp.2006.02.001

[ref43] Reis A. Nguyen V. Saheb R. Rutherford E. Sperandei S. (2023). Improving university students’ mental health literacy using experiential learning opportunities. Health Educ. J. 82, 184–199. doi: 10.1177/00178969221147615

[ref44] Ritchie J. B. Crouch G. I. (2003). The competitive destination: a sustainable tourism perspective. Wallingford, UK: Cabi.

[ref45] Rodríguez-Jiménez R.-M. Carmona M. García-Merino S. Díaz-Ureña G. Lara Bercial P. J. (2022). Embodied learning for well-being, self-awareness, and stress regulation: a randomized trial with engineering students using a mixed-method approach. Educ. Sci. 12:111. doi: 10.3390/educsci12020111

[ref46] Ryan R. M. Deci E. L. Vansteenkiste M. Soenens B. (2021). Building a science of motivated persons: self-determination theory’s empirical approach to human experience and the regulation of behavior. Motiv. Sci. 7, 97–110. doi: 10.1037/mot0000194

[ref47] Seggelen-Damen I. V. Dam K. V. (2016). Self-reflection as a mediator between self-efficacy and well-being. J. Manag. Psychol. 31, 18–33. doi: 10.1108/JMP-01-2013-0022

[ref48] Sheldon P. J. (2020). Designing tourism experiences for inner transformation. Ann. Tour. Res. 83:102935. doi: 10.1016/j.annals.2020.102935

[ref49] Shin H. H. Kim J. Jeong M. (2023). Memorable tourism experience at smart tourism destinations: do travelers' residential tourism clusters matter? Tour. Manag. Perspect. 46:101103. doi: 10.1016/j.tmp.2023.101103

[ref50] Steger M. F. Frazier P. Oishi S. Kaler M. (2006). The meaning in life questionnaire: assessing the presence of and search for meaning in life. J. Couns. Psychol. 53, 80–93. doi: 10.1037/0022-0167.53.1.80

[ref51] Sthapit E. Coudounaris D. N. (2018). Memorable tourism experiences: antecedents and outcomes. Scand. J. Hosp. Tour. 18, 72–94. doi: 10.1080/15022250.2017.1287003

[ref52] Sthapit E. Coudounaris D. N. Björk P. (2019). Extending the memorable tourism experience construct: an investigation of memories of local food experiences. Scand. J. Hosp. Tour. 19, 333–353. doi: 10.1080/15022250.2019.1689530

[ref53] Su L. Cheng J. Swanson S. (2022). The companion effect on adventure tourists’ satisfaction and subjective well-being: the moderating role of gender. Tour. Rev. 77, 897–912. doi: 10.1108/TR-02-2021-0063

[ref54] Watson D. Clark L. A. Tellegen A. (1988). Development and validation of brief measures of positive and negative affect: the PANAS scales. J. Pers. Soc. Psychol. 54, 1063–1070. doi: 10.1037/0022-3514.54.6.1063, PMID: 3397865

[ref55] Wen J. Huang S. (2019). The effects of push and pull travel motivations, personal values, and destination familiarity on tourist loyalty: a study of Chinese cigar tourists to Cuba. Asia Pacific J. Tour. Res. 24, 805–821. doi: 10.1080/10941665.2019.1635504

[ref56] Wong P. T. (2011). Positive psychology 2.0: towards a balanced interactive model of the good life. Can. Psychol. 52, 69–81. doi: 10.1037/a0022511

